# Predicting Mortality in Patients with Diabetes Starting Dialysis

**DOI:** 10.1371/journal.pone.0089744

**Published:** 2014-03-04

**Authors:** Merel van Diepen, Marielle A. Schroijen, Olaf M. Dekkers, Joris I. Rotmans, Raymond T. Krediet, Elisabeth W. Boeschoten, Friedo W. Dekker

**Affiliations:** 1 Department of Clinical Epidemiology, Leiden University Medical Center, Leiden, the Netherlands; 2 Department of Endocrinology and Metabolic Diseases, Leiden University Medical Center, Leiden, the Netherlands; 3 Department of Nephrology, Leiden University Medical Center, Leiden, the Netherlands; 4 Department of Nephrology, Academic Medical Center, Amsterdam, the Netherlands; 5 Hans Mak Institute, Naarden, the Netherlands; Universidade de São Paulo, Brazil

## Abstract

**Background:**

While some prediction models have been developed for diabetic populations, prediction rules for mortality in diabetic dialysis patients are still lacking. Therefore, the objective of this study was to identify predictors for 1-year mortality in diabetic dialysis patients and use these results to develop a prediction model.

**Methods:**

Data were used from the Netherlands Cooperative Study on the Adequacy of Dialysis (NECOSAD), a multicenter, prospective cohort study in which incident patients with end stage renal disease (ESRD) were monitored until transplantation or death. For the present analysis, patients with DM at baseline were included. A prediction algorithm for 1-year all-cause mortality was developed through multivariate logistic regression. Candidate predictors were selected based on literature and clinical expertise. The final model was constructed through backward selection. The model's predictive performance, measured by calibration and discrimination, was assessed and internally validated through bootstrapping.

**Results:**

A total of 394 patients were available for statistical analysis; 82 (21%) patients died within one year after baseline (3 months after starting dialysis therapy). The final prediction model contained seven predictors; age, smoking, history of macrovascular complications, duration of diabetes mellitus, Karnofsky scale, serum albumin and hemoglobin level. Predictive performance was good, as shown by the c-statistic of 0.810. Internal validation showed a slightly lower, but still adequate performance. Sensitivity analyses showed stability of results.

**Conclusions:**

A prediction model containing seven predictors has been identified in order to predict 1-year mortality for diabetic incident dialysis patients. Predictive performance of the model was good. Before implementing the model in clinical practice, for example for counseling patients regarding their prognosis, external validation is necessary.

## Introduction

Diabetic patients have a high risk of developing micro- and macrovascular complications such as retinopathy, (cardio)vascular disease and renal disease. According to data in the ERA-EDTA Registry, 23% of the incident end-stage renal disease (ESRD) patients had diabetes as primary renal disease [1]. Survival of diabetic dialysis patients appears inferior compared to ESRD patients without diabetes [2], [3], mainly due to cardiovascular disease [4]. Mortality in the diabetic dialysis population is high but varies significantly among patients [5], [6].

A prediction model for mortality in diabetic dialysis patients could be a helpful tool in clinical decision-making. For example, it could inform patients about their mortality risk and guide doctors and patients in their decisions on treatment. Furthermore, a prediction model that could accurately stratify patients according to their mortality risk would be useful to evaluate the composition of patients treated in a given center and provide the opportunity to compare baseline risks in comparative studies [7]. Finally, it could aid in designing a clinical trial and selecting subjects for inclusion [8]. Although some prediction models have been developed in patients with diabetes and diabetic nephropathy to predict ESRD [9]–[13], no prediction model exists in diabetic dialysis patients to predict mortality.

The primary aim of this study was to construct a prediction model to predict 1-year mortality in diabetic dialysis patients. We aimed to include easily obtainable patient characteristics, co-morbid conditions and basic laboratory variables, for the model to be convenient for clinical practice.

## Materials and Methods

### Study population

Data were collected from the Netherlands Cooperative Study on the Adequacy of Dialysis (NECOSAD), a multicenter, prospective cohort study in which 38 dialysis centers throughout the Netherlands participated. Incident adult patients were included at the start of dialysis treatment, between 1997 and 2007. Follow-up data on death were available until 2011. In the present analysis, all patients with diabetes mellitus (patients with diabetic nephropathy and patients with non- diabetic origin of ESRD but diabetes as co-morbid condition) at 3 months after the start of dialysis, which was considered the baseline of the study, were included. We chose 3 months as the start of the study for several reasons: First, at 3 months renal replacement therapy is likely to be a chronic therapy and the choice of treatment modality, hemodialysis or peritoneal dialysis, would be more definitive [14]. Furthermore, patients who recovered or died from acute renal failure within 3 months were excluded from the analysis in this way, creating a more robust model. Finally, at 3 months the clinical condition of patients is more likely to have stabilized and prognostic questions may arise at this point in time. Patients were monitored until renal transplantation or death. Informed consent was obtained before inclusion, and the Medical Ethics Committees of all participating centers approved the study (Maasstad Hospital Rotterdam, Deventer Hospital Deventer, Sint Lucas Andreas Hospital Amsterdam, Academic Medical Center Amsterdam, Maxima Medical Center Veldhoven, Catharina Hospital Eindhoven, Medical Center Haaglanden Den Haag, University Medical Center Groningen, Kennemer Gasthuis Haarlem, Atrium Medical Center Heerlen, Medical Center Leeuwarden, Leiden University Medical Center Leiden, Elisabeth Hospital Tilburg, University Medical Center Utrecht, Antonius Ziekenhuis Nieuwegein, Hospital Gelderse Vallei Ede, Haga Hospital Leyenburg Den Haag, Academic Hospital Maastricht, Jeroen Bosch Hospital Den Bosch, Medisch Spectrum Twente Enschede, Albert Schweitzer Hospital Dordrecht, Alysis Zorggroep Rijnstate Hospital Arnhem, Dianet Dialysis Center Lunetten Utrecht, Canisius Wilhelmina Hospital Nijmegen, Vie Curi Medical Center Venlo, Leveste Scheper Hospital Emmen, Dianet Dialysis Center Holendrecht Amsterdam, Haga Hospital Rode Kruis Den Haag, Rijnland Hospital Leiderdorp, Admiraal de Ruyter ziekenhuis Goes, Medical Center Alkmaar, Laurentius Ziekenhuis Roermond, Dialysis Center 't Gooi Hilversum, Groene Hart Hospital Gouda, Westfries Gasthuis Hoorn, TergooiHospitals Hilversum, Martini Ziekenhuis Groningen, Zaans Medical Center Zaandam).

### Outcome and candidate predictors

The outcome of interest was all-cause mortality within one year after inclusion (3 months). To minimize the risk of overfitting which would harm generalizability of the model and lead to poor performance in new datasets, the number of candidate predictors considered in the analysis should be limited [15], [16]. Also, decisions regarding the list of candidate predictors should be made independently of the data at hand [17]. Therefore, we composed a limited candidate predictor list a priori, that is prior to the statistical modelling process. To this end, we first made a selection of promising prognostic factors for one year mortality among incident dialysis patients with diabetes mellitus from the available variables based on a literature review. Next, we reduced this list by combining the rankings of these prognostic factors by both nephrologists and endocrinologists, resulting in the candidate predictors as described below.

Age, sex, smoking status and data on comorbidity were collected at the start of dialysis therapy. Currently smoking or smoking cessation in the three months preceding dialysis initiation was considered smoking. Comorbidity data included a history of cerebral vascular accident, myocardial infarction and peripheral vascular disease with or without amputation. At three months, laboratory values and other clinical parameters were collected. For each patient, data on diabetes mellitus were collected. To indicate the severity of diabetes mellitus, insulin-dependency, a history of diabetic retinopathy for which laser therapy was performed and patient-reported duration of diabetes mellitus were considered for the analysis. Body mass index, blood pressure and levels of hemoglobin, phosphate and serum albumin were included in the analysis. In addition, residual renal function was expressed as the residual glomerular filtration rate (rGFR), which was calculated as the mean of 24-hour creatinine and urea clearance corrected for body surface area (ml/min/1.73 m2). Finally, dialysis treatment modality (HD/PD) and the Karnofsky scale, which is a clinician-assessed scale of functional status, were included. The Karnofsky scale consists of ten levels, ranging from 10 (moribund) to 100 (normal, without limitations).

Next, the list of candidate predictors was further reduced as follows. Instead of including all selected comorbidities separately, history of cerebral vascular accident (CVA), myocardial infarction (MI) and peripheral vascular disease with amputation were combined into one predictor to indicate whether a patient had suffered from macrovascular complications. Systolic blood pressure was chosen to represent blood pressure, as this has been shown to be most predictive of mortality in dialysis patients [18]. Next, although the Karnofsky scale was registered as a categorical variable, it is of an ordinal nature and it was therefore included as a continuous variable [17]. Finally, although data on measured residual GFR (rGFR) based on 24-hour urine collection were available in NECOSAD, rGFR was not included in the main analysis since rGFR is not measured everywhere in this way as a standard procedure. Including rGFR in a prediction rule would therefore make it less practical for use in clinical practice and generalizability would be questionable. Indeed, also in the NECOSAD database a large part (18.8%) of this variable was missing. This would mean no prediction could be made for one out of every five patients. However, as most researchers and clinicians would agree rGFR could potentially be an important predictor for mortality in kidney patients and should not be overlooked a priori, we did perform an additional analysis where rGFR was included in the candidate variable list (see below). In total, these procedures resulted in a list of 14 candidate predictors for the main analysis.

### Statistical analyses

Baseline characteristics were summarized as means with standard deviations for continuous variables and as numbers with valid percentages for categorical variables, unless stated otherwise. Missing data were handled by multiple imputation methods using the fully conditional specification [19]–[21]. All predictors were imputed through linear or logistic regression as appropriate, with two exceptions: the square root of duration of DM was imputed because of non-normality, and the Karnofsky scale was imputed continuously. All candidate predictor variables were entered in a multivariate logistic regression analysis, with one-year mortality as dependent variable. Backward selection with the Akaike Information Criterion (AIC) stopping rule [17], [22] was used to identify the most significant independent predictors. In logistic regression analysis, the AIC stopping rule corresponds to a p-value<0.157 for predictor variables with one degree of freedom. Subsequently, forward selection was applied to check stability of the results. Results were pooled over imputed datasets according to Rubin's rules [23], [24].

The model's predictive performance was assessed by estimating calibration and discrimination of the model. Calibration indicates how well the model's predictions agree with the observed outcomes and was represented by the calibration slope (which is the regression coefficient of the logistic regression model with the prognostic index as the only predictor) [17], [25]. Discrimination indicates how well the model can distinguish between individuals with and without the outcome and was represented by Harrell's c-statistic (which is equal to the area under the receiver operator curve (AUC) for logistic regression analysis) [15], [17]. The apparent predictive performance, meaning the performance in the data that were used to develop the model, generally overestimates the predictive performance in new patients. Therefore, validation of the model's predictive performance is necessary to control for this potential overfitting, and internal validation was established through bootstrapping [26], [27]. The bootstrapped calibration slope was used as a shrinkage factor to adjust the model for potential overfitting and adjusted coefficients were computed [17].

To assess the robustness of the model a number of sensitivity analyses were performed by (1) checking for non-linearity of continuous variables, (2) excluding all patients with competing endpoints that were treated as alive in the original analysis, (3) including rGFR in the candidate list after imputation of missing values, (4) extending the outcome to 3-year mortality and (5) relaxing the backward selection removal criterion. Bootstrap analysis was performed using the Design package in R [28], [29] All other statistical analyses were performed using SPSS (version 20.0; SPSS Inc, Chicago, IL).

## Results

### Baseline characteristics

Baseline characteristics of the study population of diabetic incident dialysis patients (n = 394 out of a total of 2051 incident dialysis patients in NECOSAD) are shown in *Table 1*. Patients had a median age of 65 years (interquartile range 54–72) and were on average overweight (mean BMI 26.6 (5.0)). In 69% of patients the initial treatment modality was hemodialysis. Thirty-two percent of patients had macrovascular complications and 47% of patients had clinically relevant microvascular complications (retinopathy for which laser coagulation was performed). Eighty-two patients (21%) died within one year after inclusion.

**Table 1:** Baseline characteristics of the study population.

Baseline characteristics (n = 394)	
Sex (% male)	55
Age at start dialysis (median, years)	65.3 (54.4–72.4)
BMI (kg/m^2^)	26.6 (5.0)
Smoking status (current or recently quit) (%)	21
BP (mmHg)	
Systolic	149 (21)
Diastolic	78 (10)
Comorbidities (%)	
Cerebrovascular accident	13
Myocardial infarction	18
Peripheral vascular disease with amputation	5
Macrovascular complications	32
Severity of DM	
Insulin-dependency (%)	64
Duration of DM (median, years)	14 (7–22)
Retinopathy (lasercoagulation) (%)	47
Treatment modality (% HD)	69
Karnofsky scale (%)	
0–40	4
50–70	47
80–100	49
Laboratory values	
Hemoglobin (g/dl)	11.1 (1.6)
Phosphate (mmol/l)	1.8 (0.5)
Serum albumin (g/l)	34.9 (5.0)
rGFR (ml/min per 1.73 m^2^)	4.1 (2.9)

Age and duration of DM are presented as median (interquartile range). Other continuous predictors are presented as means (SD); categorical variables are presented as %.

Abbreviations: BMI, body mass index; BP, blood pressure; DM, diabetes mellitus; HD, hemodialysis; rGFR, residual glomerular filtration rate.

### Predictive variables for 1-year mortality

Fourteen candidate predictors (age, sex, BMI, smoking status, systolic blood pressure, macrovascular complications, insulin dependency, duration of diabetes, retinopathy, treatment modality, Karnofsky scale, hemoglobin level, serum phosphate and serum albumin) were included in this analysis. Percentage of missing data was on average 1.9% with a maximum of 8.9% for duration of diabetes mellitus. Five imputed datasets were created. Backward selection with the Akaike information criterion (AIC) stopping rule resulted in the final model with seven predictors; age, smoking status, Karnofsky scale, history of macrovascular complications, duration of DM, serum albumin and hemoglobin level. The pooled estimation results are presented in *Table 2*. Forward selection led to the same results, indicating stability of results.

**Table 2:** Predictive variables for 1-year mortality based on multivariate regression analysis.

Predictor	B	S.E.	P-value	B_adj
Age (years)	0.047	0.014	0.001	0.042
Smoking	0.631	0.364	0.083	0.570
Macrovascular complications	1.195	0.291	<0.001	1.078
Duration of DM (years)	0.026	0.013	0.047	0.023
Karnofsky scale	−0.043	0.010	<0.001	−0.039
Hemoglobin level (g/dl)	−0.186	0.097	0.056	−0.168
Albumin level (g/l)	−0.060	0.029	0.042	−0.054

Abbreviations: B, estimated coefficient; S.E., standard error of estimate; B_adj, estimated coefficient adjusted for overfitting.

The intercept of the model, which is necessary for computing predicted mortality risks, was 1.692 (1.610), and 1.427 when adjusted for overfitting.

All predictor variables in the final model had estimated coefficients in the expected directions. For example, smoking status had a positive coefficient, so a smoking patient has a higher probability of dying within a year. On the other hand, Karnofsky scale had a negative coefficient, so the higher the Karnofsky scale of a patient, the lower the probability of dying within a year.

To illustrate the predictions of the model, consider a non-smoking diabetic dialysis patient of 60 years old, with a previous history of myocardial infarction and a duration of diabetes mellitus of 14 years. His Karnofsky scale was 70, his Hb level was 10.5 g/dl and his albumin level was 35 g/l. This resulted in a 1-year mortality risk of 27% (95%-CI: 18%–37%). The same patient, but 10 years older and with a Karnofsky scale of only 40, would have a 1-year mortality risk of 68% (95%-CI: 51%–81%). See Table A.1 in Appendix S1 for computational details and Appendix S2 for a risk calculator.

### Validation of the model

**Figure 1:**
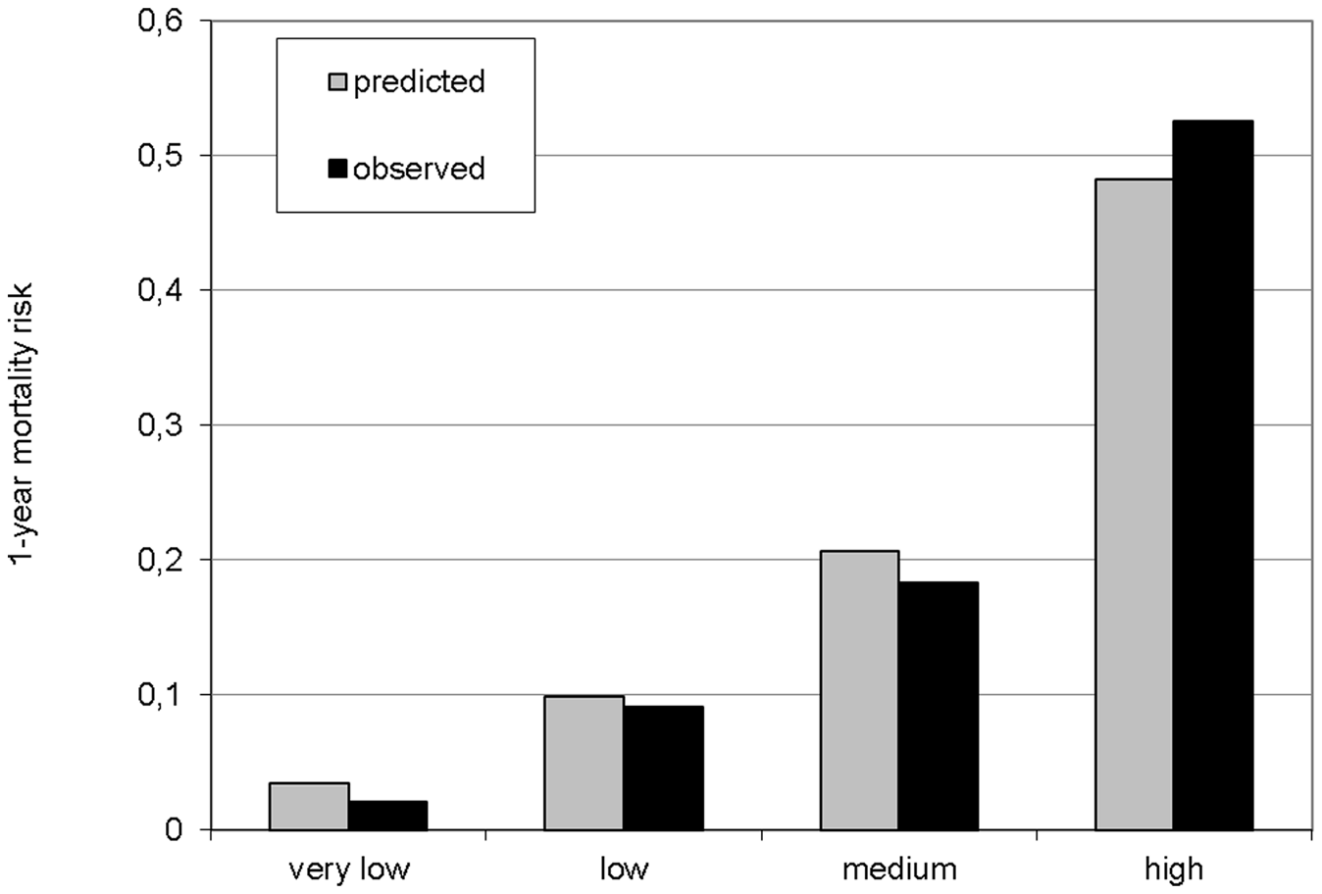
One-year mortality according to risk quartiles. Grey bars represent predicted 1-year mortality risk and black bars represent observed 1-year mortality risk.

To illustrate the calibration of the model, the model was used to predict the risk of one-year mortality for every patient in each imputed dataset used to develop the model and pooled over imputations. Then predicted mortality risk was divided into quartiles from very low to high risk, where the very low risk category represented an average risk of less than 5%, while the average predicted risk in the high risk category was about 50%. Calibration of the model was investigated by comparing observed to predicted risk across the four risk strata and is shown in *Figure 1*.

**Figure 2:**
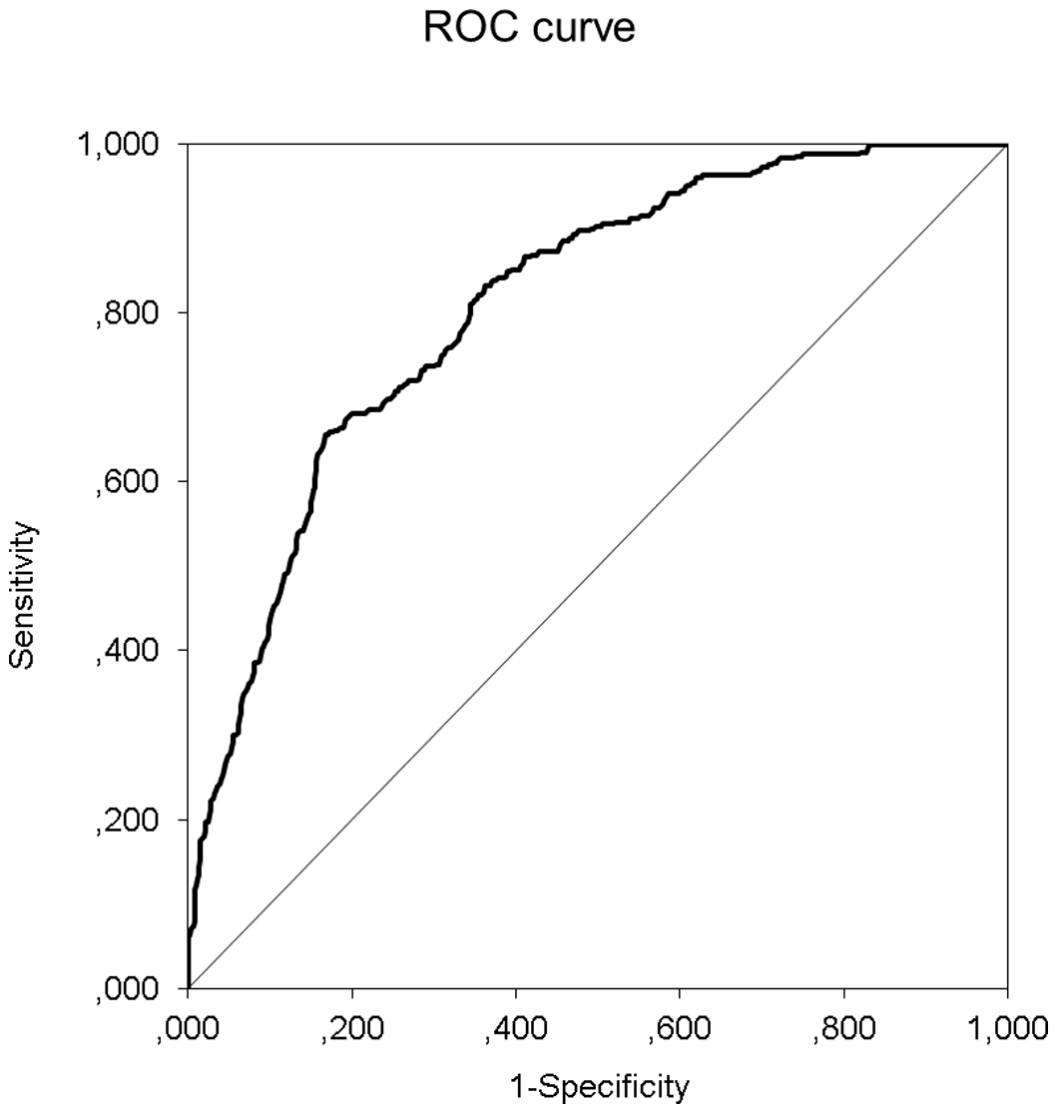
Receiver operating characteristic curve for the logistic regression model. The c-statistic was 0.810.

Discrimination was assessed by calculating the c-statistic using the receiver operator curve (ROC) for each imputed dataset and pooling results. *Figure 2* shows the ROC of the logistic regression model. The c-statistic of the model was 0.810 [0.759–0.860], indicating good discriminative ability. That is, in 81% of the cases the model will assign the highest mortality risk to a patient that dies within a year compared to a random patient that is still alive after a year.

Our model was subsequently internally validated by bootstrapping in each imputed dataset after which results were pooled. *Table 3* compares the apparent performance of the full model (before backward selection), the apparent performance of the final model, and the bootstrapped performance of the final model. The bootstrapped performance is an indication of the external performance, so how the model will perform in a new set of patients.

**Table 3:** Internal validation: apparent & optimism-corrected performance.

Performance measure	full model	final model	bootstrap
Calibration: slope	1.000	1.000	0.903
Discrimination: c-statistic	0.816	0.810	0.790

The c-statistic of the final model was 0.810, which is only slightly lower than that of the full model (0.816). Hence, the final seven predictors were able to discriminate almost as well as the total set of fourteen predictors, justifying the backward selection procedure. By definition, the calibration slope equalled 1 in the original data. After bootstrapping the calibration slope was 0.903, indicating that some overfitting was present. However, it did not substantially affect discrimination, as the c-statistic was still 0.790. For clinical purposes, the bootstrapped calibration slope estimate can be used as a shrinkage factor to compute more reliable parameter estimates, which are presented in the final column of *Table 2*.

Next, several sensitivity analyses were performed to check stability of results. First, to test for non-linearities, quadratic terms of the continuous predictors in the final model were added one by one. None of them were found significant and discrimination did not improve substantially, with a maximum c-statistic of 0.812. Second, patients with competing endpoints such as transplantation or refusal to participate within one year were treated as alive, although their actual status at one year was unknown. Excluding these patients (n = 33) from the analysis did not alter results; the same seven predictors constituted the final model with similar coefficients and model performance. Third, residual GFR was added to the candidate predictor list. In 18.8% of the cases rGFR was missing. Because of non-normality, rGFR was imputed as a square root. It was not significant on top of the seven original predictors in the final model (point estimate of −0.068 with p = 0.275), nor did it substantially improve discrimination (improvement of 0.005 in c-statistic). In an additional analysis with only patients with available rGFR, adding rGFR did not substantially improve predictive performance of the model either (improvement of 0.007 in c-statistic). As a more concise model would be preferred in clinical practice and a recent rGFR may not be available for all patients, rGFR was hence not included in the final model. Fourth, the prediction procedure was repeated for 3-year mortality. Of the 394 patients, 174 (44%) died within three years. Backward selection resulted in predominantly the same final predictor list, where smoking status and hemoglobin level in the model were replaced by sex and therapy modality. Thus, even with a broader timeframe for the outcome, the model is quite stable. The predictive performance was slightly lower, as indicated by a c-statistic of 0.784. Finally, the backwards selection removal criterion was relaxed to p-value = 0.25, which did not change the final model. Further backwards selection removal criterion to p-value = 0.50, resulted in three extra predictors in the final model (sex, BMI and insulin-dependence), but only slightly improved discrimination (c-statistic = 0.815). Since a smaller model is more convenient in practice, the final model with seven predictors was retained.

## Discussion

In this cohort study we aimed to identify predictors for 1-year all-cause mortality in diabetic patients on dialysis treatment and used the results to develop a prediction model for this population. Three hundred and ninety four incident diabetic patients were included in this analysis and 82 patients (21%) died within one year after inclusion. Candidate predictors were selected a priori based on existing literature and clinical expertise. The final prediction model contained seven predictors; age, smoking, history of macrovascular complications, duration of diabetes mellitus, Karnofsky scale, serum albumin and hemoglobin level.

Several prediction models have been developed to predict mortality in dialysis patients. Wagner et al. developed a prediction model for 3-year mortality in incident dialysis patients and found that basic patient characteristics, co-morbid conditions and laboratory values can predict 3-year mortality with a c-statistic of 0.75 [13]. Mortality in this study was somewhat lower (30%) than our 3-year mortality (44%). Holme et al. made a prediction model for total 3-year mortality in patients on hemodialysis with a c-statistic of 0.73 [30]. Mortality in this study (47%) was in line with our 3-year mortality. These prediction models included diabetes mellitus as a comorbid condition.

The current prediction model adds to existing models because it is a special model for diabetic incident dialysis patients, which includes specific diabetes-related patient characteristics and co-morbid conditions. Therefore, it is probably more accurate than existing prediction models [13], [30] in predicting mortality in this diabetic patient group, as indicated by the c-statistic of 0.810. This model will provide the opportunity to individualize treatment options. Furthermore it allows identifying and informing patients with the highest risk of death within one year. Also, as (novel) biomarkers for outcomes in this patient population are currently being developed [31], [32], an adequate basic prediction model is a requisite for assessing the additional predictive value of these biomarkers. Note that our model is not developed as a decision-tool in a pre-dialysis setting, as for such a tool, one would need both different data and different methods. Instead, our model was developed for risk stratification, i.e., to make risk predictions for new chronic dialysis patients after the clinical situation has stabilized.

There are some potential limitations in the present study. First, although the percentage of patients with diabetes in our cohort was similar to that in other European studies [5], the number of diabetic patients was relatively small for developing a prediction model. However, we controlled for potential overfitting by limiting the number of candidate predictors and bootstrapping performance measures. Second, other risk factors - such as if dialysis was started as an elective or urgent treatment, if access for dialysis was already available and social and educational variables - have been found or hypothesized to be related to mortality in diabetic or dialysis patients but have not been included in our analysis because of data restrictions. Specific examples of promising predictors lacking in the NECOSAD data are neuropathy, HbA1c level and diabetes type. Regarding neuropathy, however, adding severity of co-morbid conditions did not seem to increase their predictive power for survival in a study comparing several commonly used co-morbidity indices [33]. As for glycaemic control, we cannot exclude that difference in HbA1c level might translate into different mortality risk [34] and could improve the predictive performance of our model. Regarding diabetes type, as information on this predictor was lacking we included insulin use in our candidate predictor list, which may have been a weaker predictor. However, even without these variables, our prediction model performs well. In contrast to an etiologic study, the value of a prediction model is not judged on individual variables, but on the quality and validity of the predictions that can be made with the variables available. Predicting outcomes is different from explaining their cause [35]. All variables potentially associated with the outcome, not necessarily causally, can be considered in a prognostic study and confounding does not play a role [36], [37]. Thus, the lack of potentially important covariates in a prognostic study means there may be room for improvement of the predictive performance, but does not invalidate the current results. Because of data restrictions we could not take all mentioned risk factors into account, but it would be an interesting future research avenue to investigate whether these also contribute prognostically, and improve the predictive performance of the current model. As a third limitation, some may argue that it might be warranted to develop separate prediction models for hemodialysis and peritoneal dialysis patients. Indeed, this may result in even better predictive performance, as it could be that predictor effects differ for PD and HD patients. However, our sample size does not suffice for developing separate models or including interaction terms with therapy modality and we therefore leave this exercise to future research. And fourth, this prediction model has not been evaluated in an external data set yet, which is a necessary condition before introducing the model in clinical practice, by means of an easy to use clinical application.

Despite these limitations this prediction model is the first model that predicts mortality in diabetic incident dialysis patients with good discriminative ability, indicated by the c-statistic of 0.810. To minimize the risk of overfitting we considered a ratio of five endpoints to one candidate predictor acceptable. As larger ratios have been suggested, we additionally controlled for overfitting with internal validation through bootstrapping. Also several sensitivity analyses were performed to check robustness of the model, which showed stability of the results. For example, even a broader time frame of the predicted outcome resulted in predominantly the same final predictor list. Furthermore, the simplicity of the model with parameters that are easily to obtain makes this prediction model potentially useful for clinical practice, for example for counseling patients regarding their prognosis, and guiding doctors and patients in their decisions on future treatment.

In conclusion; a prediction algorithm for 1-year all-cause mortality has been developed for incident diabetic dialysis patients. The performance of this model is good as indicated by good outcomes for discrimination and calibration. For future research our study results need to be evaluated in an external data set. Preferentially this prediction model would be evaluated in other international and larger cohort studies before implementing in clinical practice.

## Supporting Information

Appendix S1 **Computing individual 1-year mortality risk.** Table A.1. Computation of prognostic index.
(XLS)

Appendix S2 **Risk calculator for individual 1-year mortality risk.**
(DOC)
