# Supplementary material for: Predicting Mortality in Patients with Diabetes Starting Dialysis
Source: PLoS One. 2014 Mar 4;9(3):e89744. doi: 10.1371/journal.pone.0089744 (PMC3942369; doi:10.1371/journal.pone.0089744)
Supplement: Appendix S2 — Risk calculator for individual 1-year mortality risk. (DOC) [file pone.0089744.s002.doc]

**Appendix 1: Computing individual 1-year mortality risk**

To clarify how the predicted 1-year mortality risk for a certain patient can be computed from the coefficients, consider the example of a non-smoking diabetic dialysis patient of 60 years old, with a previous history of myocardial infarction and a duration of diabetes mellitus of 14 years. His Karnofsky scale was 70, his Hb level was 10.5 g/dl and his albumin level was 35 g/l. To compute his 1-year mortality risk, his prognostic index (PI) has to be computed first. This is achieved by multiplying the estimated coefficients with the values of the predictor variables of the patient and taking the sum of these multiplications, added by the intercept of the model, see Table A.1. Now, adding all B*X terms and the intercept results in a prognostic index of -0.992. Computation of a prognostic index with the current model can also be expressed as a general formula:

PI = 1.692 + 0.047*Age (+0.631 if smoking) (+ 1.195 if macrovascular complications) + 0.026*Duration of DM - 0.043*Karnofsky scale - 0.186*Hemoglobin - 0.060*Albumin

Filling out the example values of the predictor variables results in the same value for the PI:

PI = 1.692 + 0.047*60 + 1.195 + 0.026*14 - 0.043*70 - 0.186*10.5 - 0.060*35 = 1.692 - 2.684 = -0.992.

Since the prediction model is a logistic model the predicted 1-year mortality probability can then be computed by:

1-year mortality risk = exp(PI) / (1+exp(PI)).

Thus, in this example, the 1-year mortality risk is exp(-0.992)/(1+exp(-0.992))=0.37/1.37=27%. When applying the coefficients that are adjusted for overfitting (see Table 2), the predicted 1-year mortality risk of this patient would be 26%.

**Table A.1: Computation of prognostic index**

| Predictor X | B | Value of X | B*X |
| --- | --- | --- | --- |
| Age (years) | 0.047 | 60 | 2.820 |
| Smoking | 0.631 | 0 | 0.000 |
| Macrovascular complications | 1.195 | 1 | 1.195 |
| Duration of DM (years) | 0.026 | 14 | 0.364 |
| Karnofsky scale | -0.043 | 70 | -3.010 |
| Hemoglobin level (g/dl) | -0.186 | 10.5 | -1.953 |
| Albumin level (g/l) | -0.060 | 35 | -2.100 |
| Abbreviations: B, estimated coefficient; X, predictor variable.  The intercept of the model was 1.692. | | | |
